# Genetic differentiation of spring-spawning and fall-spawning male Atlantic sturgeon in the James River, Virginia

**DOI:** 10.1371/journal.pone.0179661

**Published:** 2017-07-07

**Authors:** Matthew T. Balazik, Daniel J. Farrae, Tanya L. Darden, Greg C. Garman

**Affiliations:** 1 Center for Environmental Studies, Virginia Commonwealth University, Richmond, Virginia; 2 South Carolina Department of Natural Resources, Marine Resources Research Institute, Hollings Marine Laboratory, Charleston, South Carolina; Universidade Nova de Lisboa Instituto de Higiene e Medicina Tropical, PORTUGAL

## Abstract

Atlantic sturgeon (*Acipenser oxyrinchus oxyrinchus*, Acipenseridae) populations are currently at severely depleted levels due to historic overfishing, habitat loss, and pollution. The importance of biologically correct stock structure for effective conservation and management efforts is well known. Recent improvements in our understanding of Atlantic sturgeon migrations, movement, and the occurrence of putative dual spawning groups leads to questions regarding the true stock structure of this endangered species. In the James River, VA specifically, captures of spawning Atlantic sturgeon and accompanying telemetry data suggest there are two discrete spawning groups of Atlantic sturgeon. The two putative spawning groups were genetically evaluated using a powerful microsatellite marker suite to determine if they are genetically distinct. Specifically, this study evaluates the genetic structure, characterizes the genetic diversity, estimates effective population size, and measures inbreeding of Atlantic sturgeon in the James River. The results indicate that fall and spring spawning James River Atlantic sturgeon groups are genetically distinct (overall *F*_*ST*_ = 0.048, *F’*_*ST*_ = 0.181) with little admixture between the groups. The observed levels of genetic diversity and effective population sizes along with the lack of detected inbreeding all indicated that the James River has two genetically healthy populations of Atlantic sturgeon. The study also demonstrates that samples from adult Atlantic sturgeon, with proper sample selection criteria, can be informative when creating reference population databases. The presence of two genetically-distinct spawning groups of Atlantic sturgeon within the James River raises concerns about the current genetic assignment used by managers. Other nearby rivers may also have dual spawning groups that either are not accounted for or are pooled in reference databases. Our results represent the second documentation of genetically distinct dual spawning groups of Atlantic sturgeon in river systems along the U.S. Atlantic coast, suggesting that current reference population database should be updated to incorporate both new samples and our increased understanding of Atlantic sturgeon life history.

## Introduction

The Atlantic sturgeon (*Acipenser oxyrinchus oxyrinchus*, Acipenseridae) is an anadromous fish that currently inhabits the east coast of North America from Labrador, CA to Florida, USA [1, 2, 3, 4, 5]. Atlantic sturgeon (AS) were very abundant during European establishment of North America but were heavily exploited during the late 1800s, particularly for flesh and roe [1, 5, 6, 7, 8, 9, 10]. By the end of the 19^th^ century most of the “inexhaustible” AS populations were decimated and most fisheries collapsed [1, 5, 6, 10]; however, these populations still experienced commercial fishing pressure until the later part of the 20^th^ century when a moratorium was imposed by the Atlantic States Marine Fisheries Commission [11]. Unfortunately, as a result of AS’s long generation time in combination with continued habitat impairment such as dams and sedimentation, AS populations have been very slow to recover [4, 12, 13] which resulted in all US distinct population segments being listed as either threatened or endangered by the National Oceanic and Atmospheric Administration in 2012 [14, 15].

Previously, it was commonly accepted that AS only spawned during the spring along with most of the other anadromous fish on the east coast of the US [1, 4]. However, relatively recently researchers have shown via several lines of telemetry evidence that some rivers likely have dual-spawning groups of AS, one group spawning in the spring and another group spawning in the fall [10, 16, 17, 18]. Additionally, river-resident juvenile AS (<530 mm TL) from the Edisto River, SC were assigned to either fall or spring spawning events based on total length at day of year over a 15 year period and subsequently determined to be genetically distinct with little admixture between the spawning groups [19]. Currently dual-spawning in the James River, VA has been documented based on collections of pre- and post-spawn AS accompanied by telemetry data [10, 18]. Due to the lack of juvenile AS captures in the James River, genetics from the two spawning populations cannot be compared in the same river-resident juvenile design similar to the Edisto River study; however, adult samples are available with substantial collection information to attempt to genetically address these putative spawning groups. As such, the main objective of our study was to determine if genetic differences were present between spring and fall run AS by sampling the adult populations during the spawning seasons. Secondary objectives included determining the genetic health of the population(s) and evaluating if the use of appropriate sample selection criteria with adult AS can be used to identify dual-spawning group genetic signatures.

## Study area

The James River, river mouth located at N 36.98889, W 76.30365, is the southern-most major tributary of the Chesapeake Bay, is 696 km long and drains 26,164 km^2^. The tidal portion of the river extends up to Richmond, VA located at rkm 155. The river width varies between 0.7 and 7.1km up to rkm 120 and then narrows substantially (0.1–0.4 km). The federal navigation channel maintained by the US Army Corps of Engineers runs from the river mouth at Hampton Roads, VA to rkm 150 and is maintained to a minimum depth of 7.6 m and a minimum width of 91.4 m. AS in the James River were exploited by Europeans since the founding of Jamestown Colony in 1607 [20] and harvest continued in some capacity until 1974 when the Virginia Marine Resources Commission implemented a state-wide ban on all AS harvest [21].

## Materials and methods

### Genetic sample collection

Since October 2007, gill net sampling targeting adult AS has been conducted in the James River during the spring and fall season. Sampling protocols and tagging techniques are described in previous publications [10, 18, 22]. All sampling and fish workup was conducted following protocols set by Virginia Commonwealth University’s IACUC #AD20127 and National Oceanic and Atmospheric Administration endangered species permit #16547. From 2007 to 2016, a total of 507 unique fall run AS were captured and 40 unique AS were captured during the spring run. Fin clips were taken from most of the AS captured during the study. On occasion, fin clips for genetic samples were not taken due to high catch numbers and time constraints; however, unique passive-integrated-transponder tags were placed in every AS captured.

From the 507 fall collections, 116 males were selected for genetic analysis. The 116 (2012 n = 70, 2013 n = 46) males were picked because each had a telemetry tag surgically implanted. All fish were captured close to spawning habitat and expelled sperm during capture [10, 22]. Resulting real-time telemetry data show all but one fish routinely returned to James River spawning habitat during the fall spawning season. The one fish that did not routinely return to James River spawning habitat was captured in 2012 and was detected at the mouth of the James River during the summer of 2013 and 2014 but did not move upstream to spawning grounds. All of the 2014 (n = 2), 2015 (n = 20), and 2016 (n = 16) spring male collections (n = 38) were used for genetic analysis due to low sample size. Within the spring sample set, 18 of the 38 spring samples have accompanying telemetry data documenting repeated spring spawning movements. However, three of the non-telemetered fish captured during the spring 2015 were also recaptured in the spring 2016. All spring males either expelled sperm during capture or had sperm removed via catheter [18].

### Genetic analysis

Isolation methods, microsatellite loci, PCR conditions, visualization/scoring, and locus verification methods all followed those from Farrae et al. [19].

COLONY 2.0.6.2 was used to identify full siblings prior to analysis with *Structure* because having many closely related individuals can result in an overestimation of the number of clusters. All fish were analyzed together first with three runs using the full likelihood and pairwise-likelihood combined method (FPLS) with a polygamous breeding system, weak priors, updating allele frequencies, and no genotyping errors assumed. One run was of medium length with medium precision, one was of medium length with high precision, and one was of long length with high precision. Each spawning group was then analyzed independently with a long run length and high precision with all other parameters the same. The independent COLONY runs by spawning season were also used to estimate effective population size (*N*_*e*_) for a single time point based on sibling relationships among individuals.

The Bayesian clustering program *Structure* 2.3.4 [23] was used to observe individual genetic clustering over a range of populations (*K* varied 1–5). Run parameters were set at 10,000 burn-in repetitions followed by 10,000 Markov chain Monte-Carlo repetitions, with and without location information included as a prior, and with five independent runs per *K*. Each season also was evaluated independently under the same conditions to identify any underlying structure. Structure Harvester [24] was used to assess the results and determine the number of groups that best fit the data [25].

Genetic population structure within the James River (spring-spawning versus fall-spawning sturgeon) was evaluated using pairwise *F*_*ST*_, a measure of genetic distance, as calculated in GenAlEx 6.502 [26, 27]. *F’*_*ST*_, a measure of genetic distance that is adjusted to the maximum value of *F*_*ST*_ for a given suite of markers [28, 29], was computed for the same pairwise comparison using GenAlEx with 999 standard permutations and individual analysis suppressed.

Molecular diversity indices for the James River were calculated using GenAlEx, including number of alleles per locus (*N*_*a*_), allelic size range, and heterozygosity (*H*_*E*_) [30]. Rarified allelic richness (A; the number of alleles corrected for sample size) was calculated using FSTAT 2.9.3 [31]; inbreeding coefficients (*F*_*IS*_) [32] were calculated using GenePop 4.3 [33].

## Results and discussion

Locus *LS39* was dropped from all analyses due to substantial problems with its amplification throughout the sample set. In fall-spawning AS dataset, three loci were observed to be out of Hardy-Weinberg equilibrium (HWE): LS-68, AoxD241, and Aox12; in spring-spawning AS dataset, one locus was observed to be out of HWE: AoxD170. Given the lack of consistency in deviations from HWE and the prior use of this marker set in multiple AS studies, these loci were retained. All genotypes were checked for individual recaptures; two were identified and removed from further analysis. Three other samples could not be genotyped successfully and were excluded.

Each COLONY run produced minor variations in relatedness with 21, 22, and 18 full-sibling pairs identified in each of the three runs of the combined dataset. Individual full-sibling pairs were generally consistent between runs with the combined dataset and runs with fall and spring datasets separately. Based on the long run length and high precision (theoretically the most accurate analysis run), 17 full-sibling pairs were found in the fall-spawning dataset and 1 full-sibling pair was found in the spring-spawning dataset. Given the low number of siblings to the total sample size, these fish were not removed prior to the *Structure* analyses.

**Fig 1:**
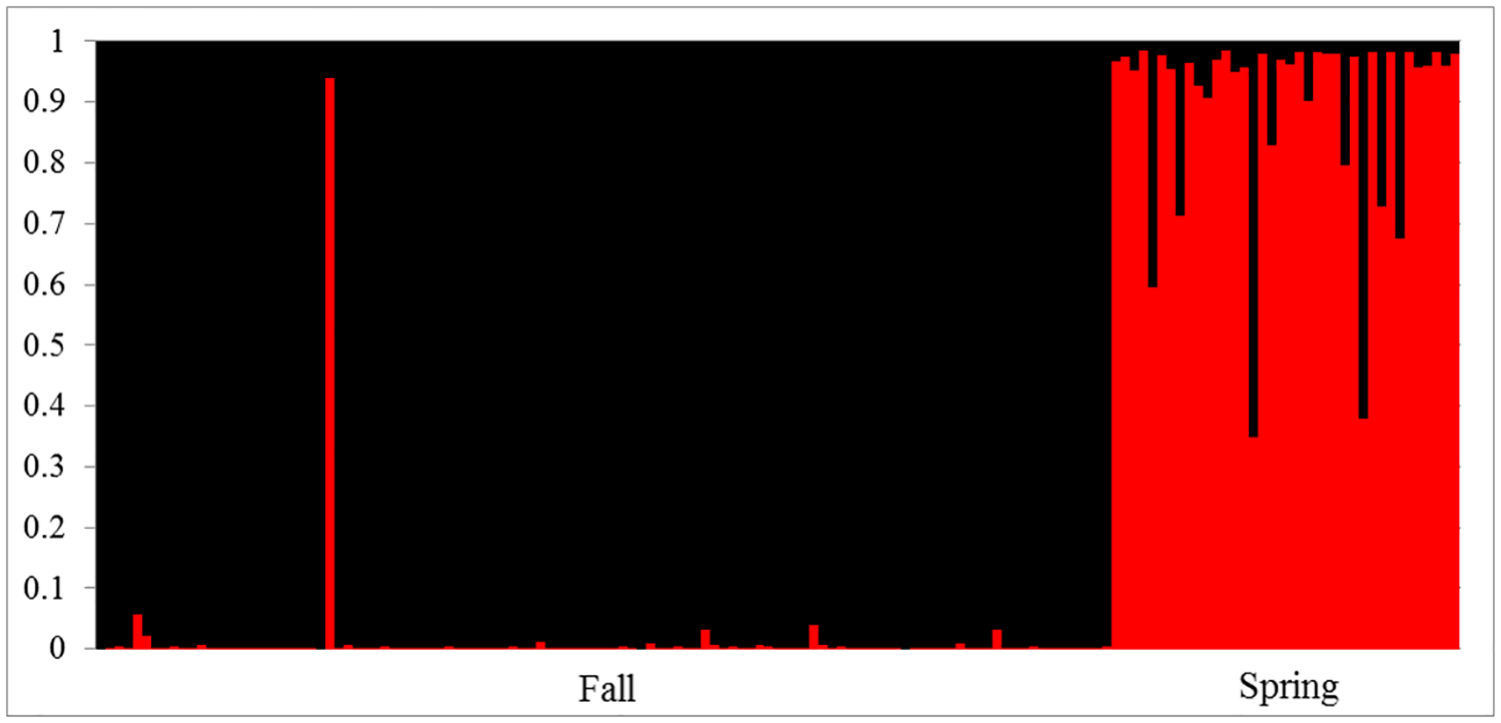
Results from *Structure* displaying the ancestry of fall spawning and spring spawning Atlantic sturgeon in the James River to *K* = 2 genetic clusters. Each individual bar represents the ancestry of a single fish, with the colors corresponding to the proportion of population ancestry.

The results from *Structure* and Structure Harvester (Δ*K* = 31.6) indicated that there were two population clusters in the James River, with distinct signatures assigned to AS from fall and spring spawning events (Fig 1). When fall and spring spawning sturgeon were evaluated independently, both resulted in *K* = 1, indicating that there was no further population or family structure present. Similar results were obtained with and without location used as a prior.

Pairwise *F*_*ST*_ between fall-spawning and spring-spawning AS was 0.048 (significant); pairwise *F’*_*ST*_ = 0.181. Heterozygosity was moderate overall for both fall (*H*_*O*_ = 0.749, *H*_*E*_ = 0.740) and spring (*H*_*O*_ = 0.708, *H*_*E*_ = 0.717) spawning groups, and no inbreeding was detected during either period (*F*_*IS*_ = -0.008 fall; *F*_*IS*_ = 0.026 spring). Effective population size was similar for both spawning AS groups in the James River (Fall: *N*_*e*_ = 46 (95% CI: 32–71), Spring: *N*_*e*_ = 44 (95% CI: 26–79)).

In the plot of results from *Structure* (Fig 1), there is a single individual in the fall cluster that appears to have a strong signature associated with the spring cluster. There are several potential explanations for this fish: it may have been mislabeled at initial collection, grouped incorrectly during genetic processing, it may represent an actual stray from the spring cluster, or it may be a stray from elsewhere that more closely resembles the spring cluster than the fall cluster. The fish was originally captured in August 2012 in close proximity to spawning habitat and expelled sperm during capture. The fish returned to the mouth of the James River in 2013 and 2014 during the summer but did not move upstream to the spawning grounds. To our knowledge, the fish has not been detected in another river. The *F*_*ST*_ and *F’*_*ST*_ results indicate that there is some gene flow between the fall and spring clusters, so it is possible that fish from one genetic cluster contribute to the opposite spawning season at times. This gene flow may also be responsible for the higher amount of admixture visible in fish in the spring cluster compared to the fall cluster, though this may also be attributed to a smaller sample size of the spring cluster.

## Conclusions

Overall, the results from *Structure* and the pairwise F_ST_ and F’_ST_ values all indicate that the fall and spring spawning adult male groups in the James River are genetically distinct. The degree of genetic differences is not as pronounced as that documented in the Edisto River [19], potentially the result of lower sample sizes from the James River or restrictions within the sample set (i.e., only adult males over a shorter collection period). The genetic diversity within both James River spawning groups are comparable to those measured in the Edisto River [19] and on the high end of genetic diversity measured in other AS populations [34, 35], with no inbreeding detected. The *N*_*e*_ estimates for both James River AS spawning populations were comparable to the estimate for the spring Edisto River population, but were an order of magnitude less than the fall Edisto River population estimate [19].

The genetics results from this study corroborate the telemetry results showing two separate spawning groups utilizing the James River [10]. Managers [2] currently recommend only river-resident AS <50 cm fork length be used for reference genetics samples to characterize populations. However, our study suggests that when eggs, larvae, or juveniles are not available for genetic analysis, spawning adults may be used as a surrogate using appropriate selection criteria, such as adult AS that expel milt or eggs during capture or fish with accompanying telemetry data that show routine returns to a certain river spawning group. Considering genetic differentiation has now been documented between spring and fall spawning AS groups in two river systems along the US Atlantic coast, careful interpretation is clearly warranted if population assignments are made assuming a single spawning population in a river, as other nearby rivers may also have dual spawning groups that either are not accounted for or are potentially pooled in reference databases. As having a biologically correct understanding of stock structure is critical to effective management and conservation, our results suggest the current reference population database should be updated to incorporate both new samples and our increased understanding of Atlantic sturgeon life history.

## Supporting information

S1 Table (TXT)
